# Sputtered Ultrathin TiO_2_ as Electron Transport Layer in Silicon Heterojunction Solar Cell Technology

**DOI:** 10.3390/nano12142441

**Published:** 2022-07-16

**Authors:** Susana Fernández, Ignacio Torres, José Javier Gandía

**Affiliations:** Departamento de Energía, CIEMAT, Avenida Complutense 40, 28040 Madrid, Spain; jj.gandia@ciemat.es

**Keywords:** titanium dioxide, magnetron sputtering, electron transport layer, silicon heterojunction solar cells

## Abstract

This work presents the implementation of ultrathin TiO_2_ films, deposited at room temperature by radio-frequency magnetron sputtering, as electron-selective contacts in silicon heterojunction solar cells. The effect of the working pressure on the properties of the TiO_2_ layers and its subsequent impact on the main parameters of the device are studied. The material characterization revealed an amorphous structure regardless of the working pressure; a rougher surface; and a blue shift in bandgap in the TiO_2_ layer deposited at the highest-pressure value of 0.89 Pa. When incorporated as part of the passivated full-area electron contact in silicon heterojunction solar cell, the chemical passivation provided by the intrinsic a-Si:H rapidly deteriorates upon the sputtering of the ultra-thin TiO_2_ films, although a short anneal is shown to restore much of the passivation lost. The deposition pressure and film thicknesses proved to be critical for the efficiency of the devices. The film thicknesses below 2 nm are necessary to reach open-circuit values above 660 mV, regardless of the deposition pressure. More so, the fill-factor showed a strong dependence on deposition pressure, with the best values obtained for the highest deposition pressure, which we correlated to the porosity of the films. Overall, these results show the potential to fabricate silicon solar cells with a simple implementation of electron-selective TiO_2_ contact deposited by magnetron sputtering. These results show the potential to fabricate silicon solar cells with a simple implementation of electron-selective TiO_2_ contact.

## 1. Introduction

Titanium dioxide (TiO_2_) is a low-cost non-toxic n-type semiconductor extensively used in a wide-range of applications such as optical and anti-reflection coatings [1,2], capacitors for microelectronic devices [3], photocatalysis [4,5], and solar cells [6,7]. This large variety of applications is feasible mainly due to its suitable optoelectronic and photocatalytic properties, its high chemical stability, and its wide band gap (~3.2 eV) [8]. Regarding its structure, TiO_2_ can exist as an amorphous layer or as one of three crystalline phases: anatase (tetragonal, a = 0.3785 nm, c = 0.9514 nm), rutile (tetragonal, a = 0.4594 nm, c = 0.2958 nm), and brookite (orthorhombic, a = 0.9184 nm, b = 0.5447 nm, and c = 0.5145 nm) [9]. It is well known that the functional properties of the TiO_2_ films are strongly dependent on the phase in which the material is found. Among them, the most stable phase is rutile, which results when preparation temperatures are above 800 °C. On the other hand, if deposition is done at temperatures below 350 °C, the obtained thin-films are generally amorphous, whereas films deposited at higher temperatures can develop the anatase phase. Finally, the brookite phase rarely develops. It is possible to exert a certain degree of control over the oxygen vacancy and the carrier concentration in order to enhance the desirable properties depending on the application field [8]. For example, doping the material with suitable metal ion dopants (Al, Nb, Sn, Ge, and Ni, among others) has proven to be an effective method for adjusting the material properties for their application into photovoltaic devices or photocatalysis and pollution sensors [10,11,12].

In the photovoltaic sector, TiO_2_ can be used in several applications, such as antireflection coating [1]. However, its role as an electron transport layers (ETL) has garnered most of the attention, with multiple examples of solar cell structures demonstrating TiO_2_’s capabilities. The halide perovskite solar cell (PSC) technology in particular has adopted mesoporous TiO_2_ films as ETL to obtain state-of-the-art solar cells. For example, PSC champions cells in [13] achieved power conversion efficiencies (PCEs) of 23.4% through suitable interfacial engineering. Dye-sensitized solar cells [6] and PSC [13,14] are typical examples were TiO_2_ is used extensively, but there are other photovoltaic technologies that also use the selective contact concept based on TiO_2,_ such as Cu(In,Ga)Se_2_ (CIGS), with examples reaching PCEs of 9.9% [15].

Low-cost and low-temperature solar cells require new device architectures that enable the use of easily available and cost-effective materials for making devices more competitive. In this sense, silicon heterojunction (SHJ) technology is compelling because its fabrication already avoids high-temperature steps while reaching record performance [16]. The development of SHJ solar cells with novel selective contacts other than the doped hydrogenated amorphous silicon (a-Si:H) layer is a current innovative concept [17]. The main advantages of this concept stem from avoiding (i) dopants, (ii) high-temperature processing, and (iii) hazardous gas precursors. Recently, ultrathin TiO_2_ layers were successfully incorporated into SHJ solar cells as a hole-blocking interface acting as ETL [18,19,20,21]. Relative to n-type c-Si, TiO_2_ has a small conduction band offset (Δ*E*_c_ ≈ 0.05 eV), which allows electrons to pass through it, and a large valence-band offset (Δ*E*_v_ ≈ 2.0 eV), which results in holes being blocked [22]. Such ETL implementation into the heterostructures offers a path towards photovoltaic technologies that use relatively simple and near-room fabrication techniques.

TiO_2_ thin films can be deposited by different techniques such as chemical vapor deposition (CVD), atomic layer deposition (ALD), dip coating, spin coating, spray pyrolysis, e-beam evaporation, and magnetron sputtering [23,24,25,26]. Among them, the method that reports the best results in the aforementioned application is ALD [24,27]. This is because it provides a uniform deposition with controllable stoichiometry and thickness, but at the expense of a very low deposition rate. Thus, regarding this last parameter, the sputtering technique can be considered a good alternative because of its potential for upscaling over large areas at considerably higher deposition rates [27,28,29]. This method has garnered great interest since it can yield either highly porous or dense films at a relatively low temperature. However, the main drawback of this technique is the damage caused at the interface during the sputtering process due to ion bombardment. This effect negatively affect the performance in the final device, and it should be minimized.

In this work, we validate the use of magnetron sputtering, a non-conventional deposition technique for the fabrication of ultrathin TiO_2_, and its application as ETL in the SHJ technology. TheTiO_2_ thin films are fabricated at room temperature (RT), varying the Argon (Ar) working pressure that aims to control the damage caused by the ion bombardment, as well as the deposition time. In this way, we successfully fabricated a collection of different solar cells and studied the effect of Ar pressure and deposition time on the main parameters, mainly the open-circuit voltage (*V*_oc_) and the fill factor (*FF*). The results successfully show the implementation of the full-area electron-selective TiO_2_ contacts into the SHJ solar device. Moreover, these results highlight the potential of the magnetron sputtering deposition technique to fabricate this kind of selective contacts for SHJ technology, opening up a new possibility to implement its large-area scale manufacturing.

## 2. Materials and Methods

We deposited the TiO_2_ thin films in a commercial UNIVEX 450B sputtering system from Leybold (Leybold GmbH, Cologne, Germany). This system is equipped with four magnetron sources, two operated by direct current (DC) and two by radio-frequency (RF). They are placed in a confocal geometry with respect to the substrate holder in order to ensure good film homogeneity. For TiO_2_ deposition, we used a 4-inch diameter TiO_2_ ceramic target provided by Neyco (Vanves, France) with a purity of 99.99% that is placed on the RF gun. The sputtering process of TiO_2_ was carried out at RT, with a base pressure of 10^−5^ Pa and in an oxygen-free environment. During the deposition, the substrate rotated at 20 r.p.m., and 9N5 Ar was used as inert gas. Its flux was controlled by an MKS mass flow controller (MKS Instruments, Andover, MA, USA) and was set to either 5 or 25 sccm, corresponding to working pressures of 0.17 Pa and 0.89 Pa, respectively. Finally, the RF power (RFP) value was set to 50 W to minimize the damage caused by ion bombardment, and the deposition time varied from 1 to 14 min.

Prior to their implementation as ETL in solar cells, 30–40 nm TiO_2_ thin films were co-deposited on resistive Corning glass (Corning Inc., New York, NY, USA) and polished n-type float zone c-Si wafers <100> to evaluate their topography and structural and optical properties. The structure of the thin films was determined with a PANalytical X’Pert Pro X-ray diffraction system (XRD), configured with a vertical Theta wide angle goniometer that uses the CuKα radiation (45 kV/40 mA). The goniometer operates in grazing incidence (GI) configuration with a Goebel-type parallel beam mirror on the incident beam side and a linear X’Celerator detector in receiving slit mode attached to a parallel plate collimator on the diffracted beam side. The patterns were obtained at a fixed incident angle of *ω* = 2.5° in parallel beam geometry and in an angular range of 20° < 2θ < 80°. The topography was evaluated using a multimode (SPM, Veeco-Digital Instruments) atomic force microscopy (AFM) in tapping mode using silicon nitride AFM tips (OTR8, Veeco). The layer roughness was quantified by the root-mean-square (RMS), determined in 5 × 5 μm^2^ 2-dimensional (2D) AFM micrographs. The chemical composition of the TiO_2_ thin films deposited onto Si substrates was determined using a JEOL JSM 7600F Schottky field emission scanning electron microscope (SEM) equipped with an “in-lens” electron detector for energy dispersive X-ray (EDX) analysis, which can be operated up to a 30 kV acceleration voltage.

The optical transmittance (*T*) spectra of the TiO_2_ films were measured at RT and normal incidence in the wavelength range 300–2500 nm with a UV/Visible/NIR PerkinElmer Lambda 1050 spectrophotometer. These spectra were used to determine the bandgap of the TiO_2_ thin films. According to the Beer–Lambert law, *T* values and the absorption coefficient (*α*) are related by the following expression (1) [30,31]:

(1) $$\alpha =\frac{1}{d}\ln\frac{1}{T}$$

where *d* is the film thickness, obtained by profilometry in previous material optimization processes. In TiO_2_ thin films, according to previously reported results, the absorption has an indirect transition (*n* = 2) [30,31]. The optical band gap energy *E*_g_ from the indirect transition can be estimated through the extrapolation of the linear part of the curve *(hνα)*^1/2^ vs *hν* given by Equation (2), to zero

(2) $${\left(h\nu \alpha \right)}^{1/2}=A\left(h\nu -E_{g}\right)$$

where *α* is estimated from (1) and *A* is a constant.

Finally, the SHJ solar cells were fabricated using 280 μm-thick double-side polished n-type float zone c-Si wafers <100> with a resistivity of 1–5 Ω·cm. The silicon wafer was dipped for 1 min in 2% HF to remove the native oxide just before loading it into a two-chamber plasma-enhanced chemical vapor deposition (PECVD) commercial reactor from Electtrorava s.p.a. (Venaria, Italy). To ensure an excellent surface passivation, 5 nm-thick intrinsic a-Si:H layers were deposited on both sides. As a hole-selective contact, 10 nm p-type a-Si:H layer was deposited on the front side of the wafer, whereas the back electron-selective-contact was performed with different thicknesses of a TiO_2_ thin film. A back full-area Al metal electrode was evaporated in a commercial UNIVEX 300 thermal evaporation system from Leybold (Leybold GmbH, Cologne, Germany), while on the front side, an 80 nm-thick indium tin oxide (ITO) transparent front contact was sputtered at RT from a In_2_O_3_:SnO_2_ (90/10 wt.%) ceramic target placed on the DC gun. To complete the solar cells, a Ti/Ag metal grid was evaporated on top of the ITO layer. The current-voltage (*IV*) characteristics of the devices were measured under illumination, calibrated at AM1.5G conditions and 100 mW/cm^2^, using a class A solar simulator (Steuernagel SC575). Transient photoconductance decay measurements were performed by using the Sinton Instrument WCT-120 (Sinton Instruments Inc., Boulder, CO, USA) to evaluate the effect of the TiO_2_ layer on the passivation layer.

## 3. Results

In the following Section 3.1, we first detail the structural, chemical, and optical characteristics of the TiO_2_ thin films deposited at two different working pressure regimes before reporting, in Section 3.2, the results on solar cells with ultra-thin TiO_2_ layer acting as ETL.

### 3.1. Characterization of TiO_2_ Layer Fabricated by Magnetron Sputtering

To characterize the structural, morphological, and optical properties of the TiO_2_ layers, 30–40 nm nominal thick thin films were deposited by RF magnetron sputtering onto glass substrates. Table 1 summarizes the sputtering deposition parameters of the TiO_2_ thin films that are considered as a reference in this work. The layers were grown without external substrate heating, and no post-process heat treatment was used.

The deposition rate varied with the Ar gas flux, decreasing when the pressure increased, as expected. The mean free path of the sputtered species (*d_m_*) and the sputtering pressure (*P*) are related to the following formula [32]:

(3) $$d_{m}=2.33{\times 10}^{-20}\frac{T}{{(P\gamma }^{2})}$$

where *T* is the temperature and *γ* is the molecular diameter. According to Equation (3), as the pressure increases, the mean free path decreases and the sputtered atoms undergo a large number of collisions, losing part of their kinetic energy. This has a several effects on the growing film. On one hand, the deposition rate decreases as seen in Table 1, due to a reduction in the rate at which the atoms reach the substrate. Furthermore, since the atoms reaching the surface also have a lower energy and thus mobility, the high deposition pressure can also influence the nucleation and growth mode of the layer, causing porosity in the microstructure [33]. On the other hand, the lower energy of the particles reaching the substrate also mean that reduced damage to the structure underneath is expected [32].

**Figure 1:**
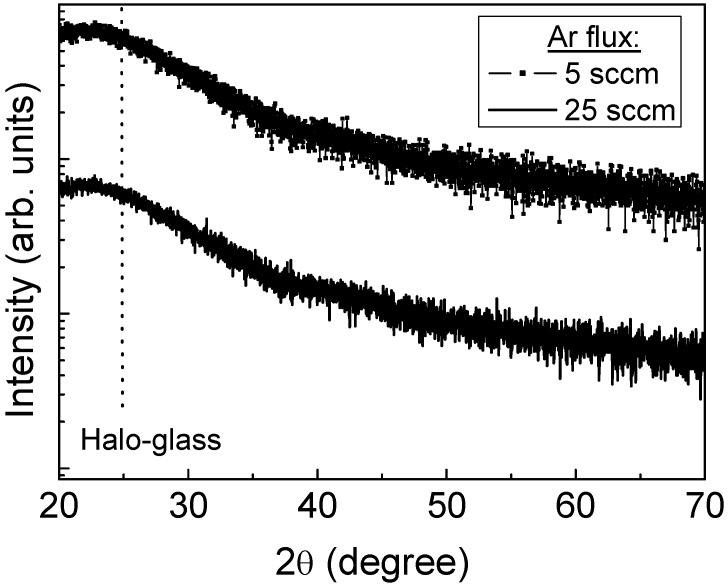
GI-XRD scans of the TiO_2_ thin films deposited on glass at different Ar gas fluxes.

Figure 1 depicts the GI-XRD scans of the samples S1 and S2. Regardless of the Ar gas flux, no characteristic peaks due to a crystalline phase appeared in the scans, indicative of films with an amorphous structure. The broad shoulder observed in the patterns around 25° corresponded to the amorphous glass. This amorphous structure is attributed to the low surface mobility that the adatoms have on the surface regardless of the sputtering conditions used because the deposition was carried out at RT.

**Figure 2:**
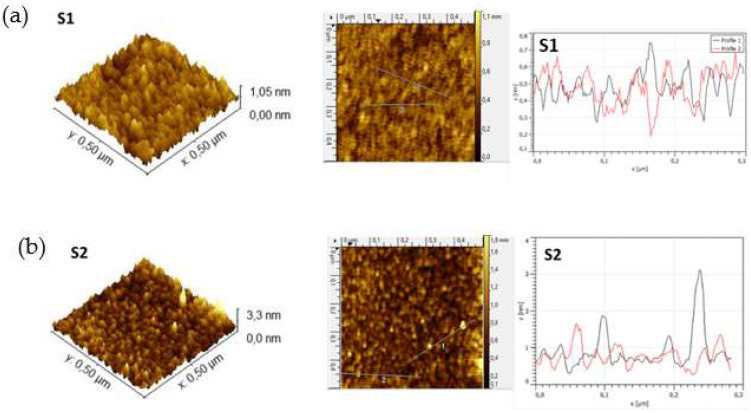
3D and 2D surface topography images of the TiO_2_ thin films deposited on glass, and profiles extracted along two different arbitrary lines on the surface at (**a**) 5 sccm (sample S1) and (**b**) 25 sccm (sample S2) Ar fluxes.

Figure 2 shows the AFM data used to evaluate the topography of the surfaces of samples S1 and S2. The 3-dimensional (3D) and 2D AFM micrographs shown in Figure 2 were taken in a scan area of 0.5 × 0.5 μm^2^. This figure also includes the profiles extracted along two different arbitrary lines on the surface. Table 2 summarizes the quantitative analysis of the roughness deduced from AFM images. The values of the average roughness (Ra), root-mean-square (RMS), and image surface area difference, defined as the difference between the image’s 3D surface area and 2D projected surface area, are included. The image surface area difference is a very useful parameter since it gives an idea about the sharpness of the surface. These parameters were obtained by processing the images with the Nanoscope analysis software 2.0. In both cases, the RMS values were in the order of Å, attributed to their amorphous nature. Both samples showed a granular morphology, although slight differences were observed depending on the working pressure: the surface of sample S1 was slightly smoother and more compact, with a very homogeneous profile across the surface, while the surface of sample S2 was rougher and more granular, with the presence of protrusions of 2–3 nm in height. The protrusions present in sample S2 are responsible for the higher image surface area difference obtained for sample S2, and its formation can be attributed to the higher probability of atom agglomeration due to the loss of mobility on the surface at higher pressures. Ultimately, the observed changes in surface morphology when the sputtering pressure was increased are indicative of a reduction in film density [33,34].

**Figure 3:**
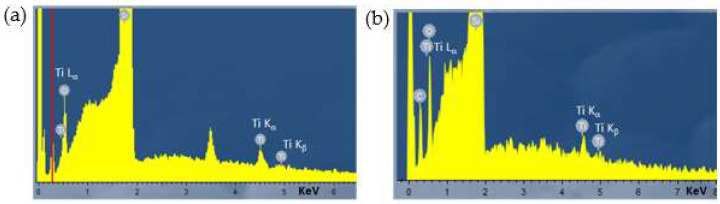
EDX spectra of TiO_2_ thin films deposited at pressures of (**a**) 0.17 Pa and (**b**) 0.8 Pa.

Chemical analysis of the as-deposited TiO_2_ layers on silicon substrate was carried out by EDX to confirm the presence of the binary oxide. Figure 3 pictures the EDX spectra acquired for the samples S1 (a) and S2 (b). The most intense peak observed at 1.74 keV came from the silicon substrate. The identified titanium (Ti) peaks were clearly observed at 4.50 and 4.93 keV, belonging to Ti K_α_ and Ti K_β_, respectively. An additional very weak Ti peak appeared at 0.39 keV, corresponding to Ti L_α_. Finally, the oxygen (O) peak was also observed at an energy of 0.52 keV (O K_α_) [35]. A very low percentage of Ti was calculated from these measurements, together with a non-stoichiometric O concentration, as is summarized in Table 3.

The large O content obtained could be in part due to [36]: (i) the extremely thin TiO_2_ layer, demonstrated by the intensity of the Si peak that dominated the spectra with respect to the peaks related to the thin film, and/or (ii) the absorption of O species such as OH and H_2_O. In this sense, the greater O content found in sample S2 suggests a higher presence of these absorbed species, mainly attributed to their higher roughness and less compact structure (see Figure 2 and Table 2), which is expected to favor the absorption of such foreign species.

**Figure 4:**
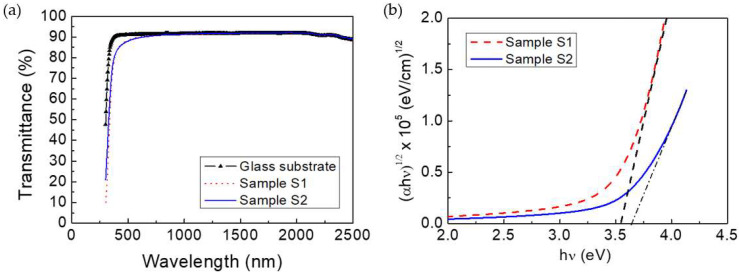
(**a**) Transmittance spectra and (**b**) Tauc’s plot for the TiO_2_ films deposited at 0.17 (red dash line) and 0.89 Pa (blue line).

The transmittance spectra of samples S1 and S2 are pictured in Figure 4a. The normalized average transmittance values at the visible wavelength range of 400–800 nm were close to 98% in both cases. This means that the slightly higher surface roughness of sample S2 did not contribute meaningfully to the light scattering. The indirect optical band gap (*E*_g_) of the TiO_2_ films was determined using the relation of Tauc’s, depicted in Figure 4b.

The *E*_g_ values obtained were 3.54 eV (sample S1) and 3.64 eV (sample S2) [9]. This blue-shift of 0.10 eV observed in sample S2 can be attributed to a combination of two effects: (i) the scattering losses because of its rougher surface, and (ii) the possible higher number of oxygen defects/vacancies created during the growth as consequence of the slower deposition rates [37]. While in the proof-of-concept cells fabricated in this work the TiO_2_ layers were deposited on the back contact, this tuning of band gap could be very beneficial for the design of rear-emitter solar cells.

### 3.2. SHJ Devices

SHJ devices with TiO_2_ films as electron-selective contacts were fabricated using the exact same conditions as those described in Section 3.1 but with different deposition times. Table 4 compiles the deposition times used, as well as the estimated thicknesses (as deduced from the deposition rates measured in thicker films) of the TiO_2_ films.

**Figure 5:**
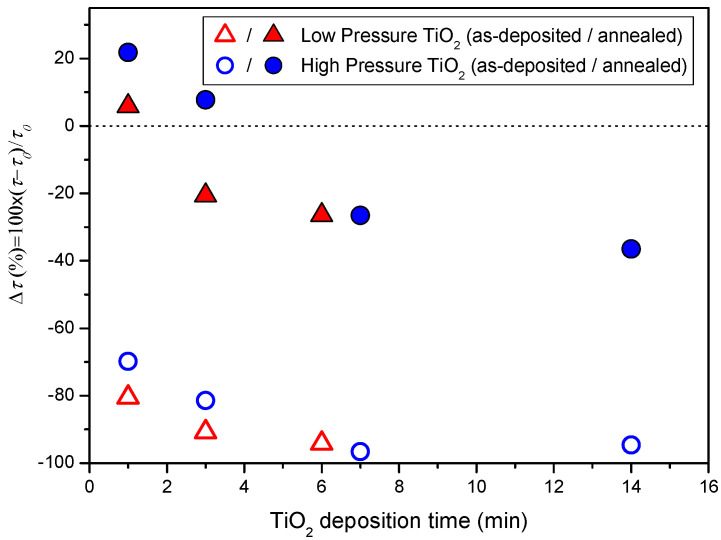
Relative change in effective minority carrier lifetime, *τ*_eff_, with respect to the value *τ*_0_ measured before TiO_2_ deposition, obtained in SHJ precursors with different TiO_2_ film thickness deposited at low pressure (red triangles) and high pressure (blue circles). Open symbols correspond to the data measured in as-deposited films, while closed symbols represent the data obtained after annealing the samples in a hot plate at 200 °C for 10 min.

Sputtering can cause damage to the underlying films not only due to ion bombardment but also due to high-energy plasma luminescence [38,39]. In the particular case of SHJ cells, UV plasma radiation contributes to a reduction of the electronic passivation provided by the a-Si:H films due to the creation of dangling bonds upon the rupture of Si-Si and Si-H bonds. Fortunately, in the case of ITO sputtering, the passivation quality can be almost fully recovered after short hot-plate annealing at temperatures compatible with the SHJ technology (i.e. 190–200 °C). To study the effect of TiO_2_ sputtering on passivation quality, the effective minority carrier lifetime (*τ*_eff_) of each sample was monitored at an excess carrier density of 10^15^ cm^−3^ before and after TiO_2_ deposition. Figure 5 shows the relative changes in *τ*_eff_ with respect to the value *τ*_0_ measured before TiO_2_ deposition (but after front ITO deposition and a subsequent anneal to recover de damage due to ITO sputtering) in the as-deposited stage and after a 10 min anneal in a hot-plate at 200 °C.

As can be seen from the as-deposited results, the passivation quality drops drastically even after 1 min plasma. Further increasing the deposition causes further degradation of the passivation, but at a much lower pace. For all the data gathered, the degradation is slightly lower in the high-pressure regime. Since both ion bombardment and plasma luminescence are responsible to the degradation caused by sputtering processes, it appears that the higher pressure does indeed limit ion bombardment to an extent.

**Figure 6:**
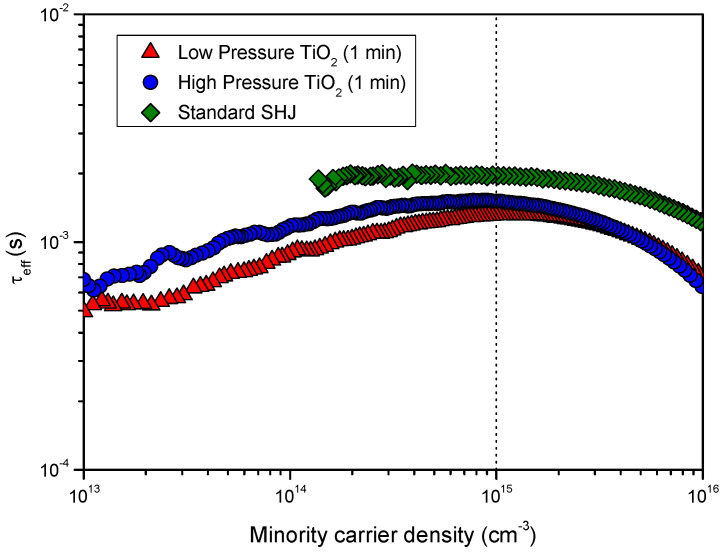
Injection-level-dependent effective lifetimes of the SHJ precursors with the electron selective contact consisting of a thin intrinsic a-Si:H layer capped with a TiO_2_ film deposited in the low-pressure regime or high-pressure regime. For comparison, data for a standard SHJ cell precursor (with n-doped a-Si:H as the electron selective layer) are also included.

By annealing the samples for 10 min at 200 °C, *τ*_eff_ increases from the as-deposited state, as occurs after ITO deposition. The relative recovery is higher for the films deposited in the high-pressure regime, which we attributed to the lower damage produced by the passivation when using the high-pressure regime. For the higher deposition times, the short anneal does not fully recover the passivation regardless of the pressure regime, implying that the sputtering damage is not entirely reverted. However, for deposition times lower than 3 min, in the case of the high-pressure regime, and 1 min, in the case of the low-pressure regime, the measured *τ*_eff_ shows an improvement of up to 20% over the samples with only a-Si:H and ITO. This improvements at the lowest deposition times hints at a field-effect passivation on top of the chemical passivation provided by the a-Si:H films [40]. Nevertheless, since the effect is not sustained over thicker films, it is clear that it is not strong enough to counteract the damage generated but for the thinnest films. As an example, Figure 6 shows *τ*_eff_ measured for the 1 min TiO_2_ films deposited in both pressure regimes as well as for a standard SHJ device with n-doped a-Si:H film at the electron contact. The better field effect passivation provided by the n-doped a-Si:H layer is apparent in these results since *τ*_eff_ is slightly better for the standard SHJ structure.

**Figure 7:**
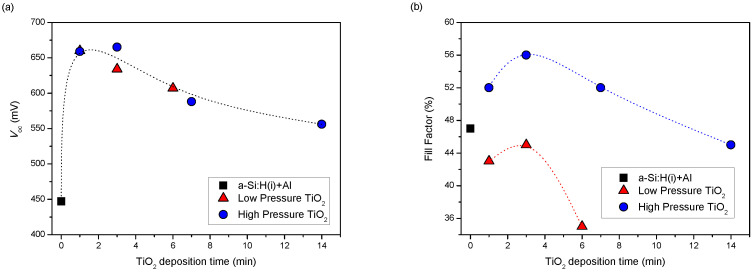
(**a**) *V*_oc_ and (**b**) *FF* values of the solar cells fabricated with TiO_2_ layers with different thicknesses (deposition time) deposited in the two pressure regimes studied. Data for the control cell with the aluminum back contact directly evaporated onto the a-Si:H(i) layer is also shown for comparison (lines are included as a guide to the eye).

Following the TiO_2_ deposition, the devices were completed by evaporating a full area aluminum back contact and a silver grid onto the front ITO. Additionally, a control cell with the aluminum back contact directly evaporated onto the a-Si:H(i) layer was fabricated for comparison. The solar cells were then measured under calibrated illumination and the solar cells parameters (*FF*, *V*_oc_, *J*_sc_) extracted. Figure 7 displays the values of *FF* and *V*_oc_ as a function of TiO_2_ deposition time for the solar cells studied. We did not observe any meaningful variations with TiO_2_ in *J*_sc_ and have thus omitted the data from Figure 7.

Even though the TiO_2_ films deposited in the high-pressure regime showed improved surface passivation, the *V*_oc_ in the final devices did not depend on the deposition regime. Instead, *V*_oc_ depended solely on the TiO_2_ thickness/deposition time, as shown in Figure 7. *V*_oc_ reached a maximum of 660-665 mV after 1–2 nm of TiO_2_ (1–3 min depositing time) covered the a-Si:H(i) layer and then declined progressively for thicker films. *V*_oc_ follows a similar trend to that of *τ*_eff_ for thicker films and hence we can apply the same arguments. As the deposition time increases, the damage due to the sputtering process generates recombination centers that do not revert upon annealing and degrades the passivation and the measured *V*_oc_. On the other hand, the measured *V*_oc_ values when TiO_2_ is used are all considerably higher than when Al is directly deposited onto the a-Si:H(i) layer. We can thus hypothesize that, even though the AFM analysis of thicker films indicated a decrease in film density with increasing deposition pressure, both deposition regimes yield TiO_2_ films sufficiently dense as to block the majority of the incoming Al atoms from reaching the a-Si:H(i) layer and the c-Si surface. Otherwise, the passivation would drastically deteriorate, which does not seems to be the case from the *V*_oc_ values obtained [41]. Finally, is worth pointing out that although the measured *V*_oc_ is lower than what is typically obtained in standard SHJ solar cells (>700 mV), this is in line with the results obtained for passivated TiO_2_ contacts deposited by ALD [42]. 

**Figure 8:**
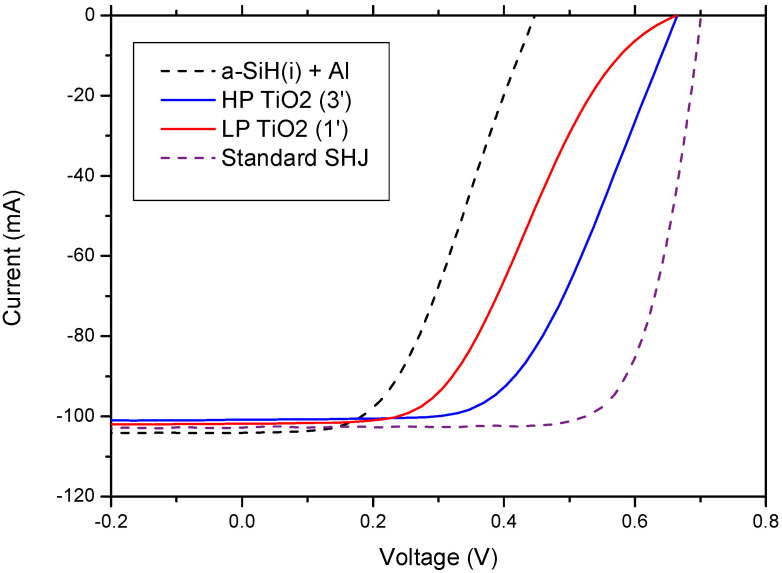
Current-voltage characteristics of the best solar cells fabricated with TiO_2_ films deposited in the two pressure regimes studied. For comparison, characteristics of the control solar cell with the aluminum back contact directly evaporated onto the a-Si:H (i) layer and a standard SHJ cell are also included.

The deposition pressure has a clear effect on the *FF* though. TiO_2_ films deposited at 0.89 Pa yield solar cells with consistently higher *FF* than cells with TiO_2_ deposited at 0.17 Pa. Furthermore, cells with TiO_2_ deposited in the high-pressure regime also present a higher *FF* than the control cell (as long as the TiO_2_ thickness is below ~7 nm, i.e., below 14 min deposition time), while cells with TiO_2_ deposited in the low-pressure regime show *FF* values systematically lower than the control cell. Interestingly, the *IV* curves of the cells with low-pressure-deposited TiO_2_ all display a marked S-shaped curve. On the other hand, the *IV* curves obtained for the cells with high-pressure-deposited TiO_2_ behave normally or have a much less pronounced S-shape. As an example, Figure 8 pictures the *IV* characteristics of the best solar cells obtained with TiO_2_ as the electron-selective contact, as well as the characteristics of the control cell and a standard SHJ cell fabricated with similar a-Si:H layers.

The large influence of deposition pressure on the *IV* characteristics must be related to the differences in TiO_2_ thin films properties. The data obtained from the material characterization showed that TiO_2_ films deposited in the high-pressure regime had a higher porosity. In addition, the modest *FF* values measured in the fabricated solar cells suggest very high resistivity for these films. Higher porosity would facilitate the incorporation of Al atoms inside the film during the contact formation, creating parallel low-resistivity paths, reducing the overall film resistivity and explaining the trend in *FF* with deposition pressure and film thickness.

## 4. Conclusions

This works aims to validate magnetron sputtering as a deposition technique to fabricate ultrathin TiO_2_ layers as selective contacts for silicon heterojunction solar cell technology. Prior to the incorporation of the thin films as full-area ETL into the solar cell, the main properties of 30–40 nm-thick TiO_2_ films, fabricated at room temperature and at different working pressures, were determined. The results show amorphous TiO_2_ thin films, with a morphology clearly determined by the effect of pressure. When implementing the TiO_2_ into a device, good control of both the pressure and the deposition time is essential to minimize the damage caused to it. A very thin TiO_2_ layer is essential to maintain the passivation quality provided by the intrinsic a-Si:H film, and high deposition pressure is found to be decisive in order to reach the best *FF*. These results validate the potential of such deposition techniques to fabricate full-area selective contacts in silicon solar cell technology, thanks to the proper optimization of the pressure and the deposition time. Thus, it could be very profitable to incorporate them into large-area scale manufacture.

## Figures and Tables

**Table 1:** Deposition parameters used for the sputtering of the TiO_2_ layers.

Sample	Ar Flux (sccm)	Pressure (Pa)	RFP (W)	Deposition Time (min)	Rate (nm/min)
S1	5	0.17	50	60	0.70
S2	25	0.89	50	60	0.55

**Table 2:** Average roughness Ra, root-mean-square RMS, and image surface area difference of the sputtered TiO_2_ layers.

Sample	Ra (Å)	RMS (Å)	Image Surface Area Difference (%)
S1	94.7	119	0.088
S2	195	257	0.332

**Table 3:** Chemical analysis from EDX.

Sample	Ti (%at)	O (%at)	Ti:O Ratio
S1	0.33	4.82	0.07
S2	0.37	6.10	0.06

**Table 4:** Deposition times and estimated thicknesses of the TiO_2_ films used as electron selective contacts in silicon heterojunction solar cells. The deposition rates used for estimating the thicknesses were 0.73 nm/min and 0.55 nm/min for the low-pressure and for the high-pressure regime, respectively.

**Regime**	**Deposition Time (min)**	**Estimated Thickness (nm)**
Low-pressure (0.17 Pa)	1	0.7
	3	2.2
	6	4.4
High-pressure (0.89 Pa)	1	0.6
	3	1.7
	7	3.8
	14	7.7

## Data Availability

Not applicable.

## References

[1] Afzal A., Habib A., Ulhasan I., Shahid M., Rehman A. (2021). Antireflective Self-Cleaning TiO_2_ Coatings for Solar Energy Harvesting Applications. Front. Mater..

[2] Salvaggio M.G., Passalacqua R., Abate S., Perathoner S., Centi G., Lanza M., Stassi A. (2016). Functional nano-textured titania-coatings with self-cleaning and antireflective properties for photovoltaic surfaces. Sol. Energy.

[3] Gyanan, Mondal S., Kumar A. (2016). Tunable dielectric properties of TiO_2_ thin film based MOS systems for application in microelectronics. Superlattices Microstruct..

[4] Fujishima A., Zhang X., Tryk D. (2008). TiO_2_ photocatalysis and related surface phenomena. Surf. Sci. Rep..

[5] Basavarajappa P.S., Patil S.B., Ganganagappa N., Reddy K.R., Raghu A.V., Reddy C.V. (2020). Recent progress in metal-doped TiO_2_, non-metal doped/codoped TiO_2_ and TiO_2_ nanostructured hybrids for enhanced photocatalysis. Int. J. Hydrog. Energy.

[6] Aboulouard A., Gultekin B., Can M., Erol M., Jouaiti A., Elhadadi B., Zafer C., Demic S. (2020). Dye sensitized solar cells based on titanium dioxide nanoparticles synthesized by flame spray pyrolysis and hydrothermal sol-gel methods: A comparative study on photovoltaic performances. J. Mater. Res. Technol..

[7] Dubey R.S., Jadkar S.R., Bhorde A.B. (2021). Synthesis and Characterization of Various Doped TiO_2_ Nanocrystals for Dye-Sensitized Solar Cells. ACS Omega.

[8] Lourduraj S. (2020). Titanium Dioxide Versatile Solid Crystalline: An Overview. Assorted Dimensional Reconfigurable Materials.

[9] Poddar N.P., Mukherjee S. (2016). Characterization of TiO_2_ thin films deposited by using dc magnetron sputtering. Carbon Sci. Technol..

[10] Alem A., Sarpoolaky H. (2010). The effect of silver doping on photocatalytic properties of titania multilayer membranes. Solid State Sci..

[11] Athira K., Merin K.T., Raguram T., Rajni K.S. (2020). Synthesis and characterization of Mg doped TiO_2_ nanoparticles for photocatalytic applications. Mater. Today Proc..

[12] Waghchaure R.H., Koli P.B., Adole V.A., Pawar T.B., Jagdale B.S. (2021). Transition metals Fe^3+^, Ni^2+^ modified titanium dioxide (TiO_2_) film sensors fabricated by CPT method to sense some toxic environmental pollutant gases. J. Indian Chem. Soc..

[13] Seo S., Shin S., Kim E., Jeong S., Park N.-G., Shin H. (2021). Amorphous TiO_2_ Coatings Stabilize Perovskite Solar Cells. ACS Energy Lett..

[14] Homola T., Pospisil J., Shekargoftar M., Svoboda T., Hvojnik M., Gemeiner P., Weiter M., Dzik P. (2020). Perovskite Solar Cells with Low-Cost TiO_2_ Mesoporous Photoanodes Prepared by Rapid Low-Temperature (70 °C) Plasma Processing. ACS Appl. Energy Mater..

[15] Hsu W., Sutter-Fella C.M., Hettick M., Cheng L., Chan S., Chen Y., Zeng Y., Zheng M., Wang H.P., Chiang C.C. (2015). Electron-Selective TiO_2_ Contact for Cu(In,Ga)Se2 Solar Cells. Sci. Rep..

[16] Green M.A., Dunlop E.D., Hohl-Ebinger J., Yoshita M., Kopidakis N., Hao X. (2021). Solar cell efficiency tables (Version 58). Prog. Photovolt. Res. Appl..

[17] Nagamatsu K.A., Avasthi S., Sahasrabudhe G., Man G., Jhaveri J., Berg A.H., Schwartz J., Kahn A., Wagner S., Sturm J.C. (2015). Titanium dioxide/silicon hole-blocking selective contact to enable double-heterojunction crystalline silicon-based solar cell. Appl. Phys. Lett..

[18] Lee Y.-T., Lin F.-R., Pei Z. (2020). Solution-Processed Titanium Oxide for Rear Contact Improvement in Heterojunction Solar Cells. Energies.

[19] Boccard M., Yang X., Weber K., Holman Z.C. Passivation and carrier selectivity of TiO_2_ contacts combined with different passivation layers and electrodes for silicon solar cells. Proceedings of the 2016 IEEE 43rd Photovoltaic Specialists Conference (PVSC).

[20] He J., Ling Z., Gao P., Ye J. (2017). TiO_2_ Films from the Low-Temperature Oxidation of Ti as Passivating-Contact Layers for Si Heterojunction Solar cells. Sol. RRL.

[21] Li F., Sun Z., Zhou Y., Wang Q., Zhang Q., Dong G., Liu F., Fan Z., Liu Z., Cai Z. (2019). Lithography-free and dopant-free back-contact silicon heterojunction solar cells with solution-processed TiO_2_ as the efficient electron selective layer. Sol. Energy Mater. Sol. Cells.

[22] Avasthi S., McClain W.E., Man G., Kahn A., Schwartz J., Sturm J.C. (2013). Hole-blocking titanium-oxide/silicon heterojunction and its application to photovoltaics. Appl. Phys. Lett..

[23] Matkivskyi V., Lee Y., Seo H.S., Lee D.-K., Park J.-K., Kim I. (2021). Electronic-beam evaporation processed titanium oxide as an electron selective contact for silicon solar cells. Curr. Appl. Phys..

[24] Masmitjà G., Ros E., Almache-Hernández R., Pusay B., Martín I., Voz C., Saucedo E., Puigdollers J., Ortega P. (2022). Interdigitated back-contacted crystalline silicon solar cells fully manufactured with atomic layer deposited selective contacts. Sol. Energy Mater. Sol. Cells.

[25] Saheed M.S.M., Mohamed N.M., Singh B.S.M., Perumal V., Saheed M.S.M. DC magnetron sputtered TiO_2_ thin film as efficient hole blocking layer for perovskite solar cell. Proceedings of the 2017 IEEE Regional Symposium on Micro and Nanoelectronics (RSM).

[26] Chen Z., Dündar I., Oja Acik I., Mere A. (2019). TiO_2_ thin films by ultrasonic spray pyrolysis. IOP Conf. Ser. Mater. Sci. Eng..

[27] Zhu H., Zhang T.-h., Wei Q.-y., Yu S.-j., Gao H., Guo P.-c., Li J.-k., Wang Y.-x. (2022). Preparation of TiO_2_ electron transport layer by magnetron sputtering and its effect on the properties of perovskite solar cells. Energy Rep..

[28] Chen C., Cheng Y., Dai Q., Song H. (2015). Radio Frequency Magnetron Sputtering Deposition of TiO_2_ Thin Films and Their Perovskite Solar Cell Applications. Sci. Rep..

[29] Zhang M., Chen J., Xuan W., Song X., Xu H., Zhang J., Wu J., Jin H., Dong S., Luo J. (2021). Comparison of sputtering and atomic layer deposition based ultra-thin alumina protective layers for high temperature surface acoustic wave devices. J. Mater. Res. Technol..

[30] Rahman K.H., Kar A.K. (2019). Effect of precursor concentration of microstructured titanium-di-oxide (TiO_2_) thin films and their photocatalytic activity. Mater. Res. Express.

[31] López R., Gómez R. (2011). Band-gap energy estimation from diffuse reflectance measurements on sol–gel and commercial TiO_2_: A comparative study. J. Sol-Gel Sci. Technol..

[32] Dave V., Dubey P., Gupta H.O., Chandra R. (2013). Influence of sputtering pressure on the structural, optical and hydrophobic properties of sputtered deposited HfO_2_ coatings. Thin Solid Film..

[33] Eufinger K., Janssen E.N., Poelman H., Poelman D., De Gryse R., Marin G.B. (2006). The effect of argon pressure on the structural and photocatalytic characteristics of TiO_2_ thin films deposited by d.c. magnetron sputtering. Thin Solid Film..

[34] Wang Y.-H., Rahman K.H., Wu C.-C., Chen K.-C. (2020). A Review on the Pathways of the Improved Structural Characteristics and Photocatalytic Performance of Titanium Dioxide (TiO_2_) Thin Films Fabricated by the Magnetron-Sputtering Technique. Catalysts.

[35] Augustowski D., Kwaśnicki P., Dziedzic J., Rysz J. (2020). Magnetron Sputtered Electron Blocking Layer as an Efficient Method to Improve Dye-Sensitized Solar Cell Performance. Energies.

[36] Hotový I., Pullmannová A., Predanocy M., Hotový J., Rehácek V., Kups T., Spiess L. (2009). Structural and morphological investigations of TiO_2_ sputtered thin film. J. Electr. Eng..

[37] Shei S.-C. (2013). Optical and Structural Properties of Titanium Dioxide Films from and Starting Materials Annealed at Various Temperatures. Adv. Mater. Sci. Eng..

[38] Demaurex B., De Wolf S., Descoeudres A., Charles Holman Z., Ballif C. (2012). Damage at hydrogenated amorphous/crystalline silicon interfaces by indium tin oxide overlayer sputtering. Appl. Phys. Lett..

[39] Le A.H.T., Dao V.A., Pham D.P., Kim S., Dutta S., Thi Nguyen C.P., Lee Y., Kim Y., Yi J. (2019). Damage to passivation contact in silicon heterojunction solar cells by ITO sputtering under various plasma excitation modes. Sol. Energy Mater. Sol. Cells.

[40] Yang X., Bi Q., Ali H., Davis K., Schoenfeld W.V., Weber K. (2016). High-Performance TiO_2_-Based Electron-Selective Contacts for Crystalline Silicon Solar Cells. Adv. Mater..

[41] Li S., Pomaska M., Lambertz A., Duan W., Bittkau K., Qiu D., Yao Z., Luysberg M., Steuter P., Köhler M. (2021). Transparent-conductive-oxide-free front contacts for high-efficiency silicon heterojunction solar cells. Joule.

[42] Acharyya S., Sadhukhan S., Panda T., Ghosh D.K., Mandal N.C., Nandi A., Bose S., Das G., Maity S., Chaudhuri P. (2022). Dopant-free materials for carrier-selective passivating contact solar cells: A review. Surf. Interfaces.

